# Supplementary material for: HIVprotI: an integrated web based platform for prediction and design of HIV proteins inhibitors
Source: J Cheminform. 2018 Mar 9;10:12. doi: 10.1186/s13321-018-0266-y (PMC5845081; doi:10.1186/s13321-018-0266-y)
Supplement: Supplementary file 2 — Additional file 2. Source code of HIVProtI web server. [file 13321_2018_266_MOESM2_ESM.zip › HIVprotI_Source-code/hivprotI/weka-3-6-6/doc/weka/core/ProtectedProperties.html]

ProtectedProperties


---


|  |  |  |
| --- | --- | --- |
| **Overview** \| **Package** \| **Class** \| **Tree** \| **Deprecated** \| **Index** \| **Help** \| **Weka's home** | |  |
| **PREV CLASS**   **NEXT CLASS** | **FRAMES**    **NO FRAMES**     **All Classes** |  |
| SUMMARY: NESTED \| FIELD \| CONSTR \| METHOD | DETAIL: FIELD \| CONSTR \| METHOD |  |


---


## weka.core Class ProtectedProperties

```
java.lang.Object
  java.util.Dictionary<K,V>
      java.util.Hashtable<java.lang.Object,java.lang.Object>
          java.util.Properties
              weka.core.ProtectedProperties
```

**All Implemented Interfaces:**: java.io.Serializable, java.lang.Cloneable, java.util.Map<java.lang.Object,java.lang.Object>, RevisionHandler

---

``` public class ProtectedProperties extends java.util.Properties implements RevisionHandler ```

Simple class that extends the Properties class so that the properties are
unable to be modified.

**Version:**
:   $Revision: 1.6 $

**Author:**
:   Richard Kirkby (rkirkby@cs.waikato.ac.nz)

**See Also:**: Serialized Form

---

**Constructor Summary**

`ProtectedProperties(java.util.Properties props)`
  
          Creates a set of protected properties from a set of normal ones.


| **Method Summary** | |
| --- | --- |
| `void` | `clear()`             Overrides a method to prevent the properties from being modified. |
| `java.lang.String` | `getRevision()`             Returns the revision string. |
| `void` | `load(java.io.InputStream inStream)`             Overrides a method to prevent the properties from being modified. |
| `java.lang.Object` | `put(java.lang.Object key, java.lang.Object value)`             Overrides a method to prevent the properties from being modified. |
| `void` | `putAll(java.util.Map t)`             Overrides a method to prevent the properties from being modified. |
| `java.lang.Object` | `remove(java.lang.Object key)`             Overrides a method to prevent the properties from being modified. |
| `java.lang.Object` | `setProperty(java.lang.String key, java.lang.String value)`             Overrides a method to prevent the properties from being modified. |

**Methods inherited from class java.util.Properties**

`getProperty, getProperty, list, list, load, loadFromXML, propertyNames, save, store, store, storeToXML, storeToXML, stringPropertyNames`

**Methods inherited from class java.util.Hashtable**

`clone, contains, containsKey, containsValue, elements, entrySet, equals, get, hashCode, isEmpty, keys, keySet, size, toString, values`

**Methods inherited from class java.lang.Object**

`getClass, notify, notifyAll, wait, wait, wait`

**Constructor Detail**

### ProtectedProperties

```
public ProtectedProperties(java.util.Properties props)
```

:   Creates a set of protected properties from a set of normal ones.

    **Parameters:**: `props` - the properties to be stored and protected.


**Method Detail**

### setProperty

```
public java.lang.Object setProperty(java.lang.String key,
                                    java.lang.String value)
```

:   Overrides a method to prevent the properties from being modified.

    :   **Overrides:**: `setProperty` in class `java.util.Properties`
    :   **Returns:**: never returns without throwing an exception. **Throws:**: `java.lang.UnsupportedOperationException` - always.

---


### load

```
public void load(java.io.InputStream inStream)
```

:   Overrides a method to prevent the properties from being modified.

    :   **Overrides:**: `load` in class `java.util.Properties`
    :   **Throws:**: `java.lang.UnsupportedOperationException` - always.

---


### clear

```
public void clear()
```

:   Overrides a method to prevent the properties from being modified.

    :   **Specified by:**: `clear` in interface `java.util.Map<java.lang.Object,java.lang.Object>` **Overrides:**: `clear` in class `java.util.Hashtable<java.lang.Object,java.lang.Object>`
    :   **Throws:**: `java.lang.UnsupportedOperationException` - always.

---


### put

```
public java.lang.Object put(java.lang.Object key,
                            java.lang.Object value)
```

:   Overrides a method to prevent the properties from being modified.

    :   **Specified by:**: `put` in interface `java.util.Map<java.lang.Object,java.lang.Object>` **Overrides:**: `put` in class `java.util.Hashtable<java.lang.Object,java.lang.Object>`
    :   **Returns:**: never returns without throwing an exception. **Throws:**: `java.lang.UnsupportedOperationException` - always.

---


### putAll

```
public void putAll(java.util.Map t)
```

:   Overrides a method to prevent the properties from being modified.

    :   **Specified by:**: `putAll` in interface `java.util.Map<java.lang.Object,java.lang.Object>` **Overrides:**: `putAll` in class `java.util.Hashtable<java.lang.Object,java.lang.Object>`
    :   **Throws:**: `java.lang.UnsupportedOperationException` - always.

---


### remove

```
public java.lang.Object remove(java.lang.Object key)
```

:   Overrides a method to prevent the properties from being modified.

    :   **Specified by:**: `remove` in interface `java.util.Map<java.lang.Object,java.lang.Object>` **Overrides:**: `remove` in class `java.util.Hashtable<java.lang.Object,java.lang.Object>`
    :   **Returns:**: never returns without throwing an exception. **Throws:**: `java.lang.UnsupportedOperationException` - always.

---


### getRevision

```
public java.lang.String getRevision()
```

:   Returns the revision string.

    :   **Specified by:**: `getRevision` in interface `RevisionHandler`
    :   **Returns:**: the revision


---


|  |  |  |
| --- | --- | --- |
| **Overview** \| **Package** \| **Class** \| **Tree** \| **Deprecated** \| **Index** \| **Help** \| **Weka's home** | |  |
| **PREV CLASS**   **NEXT CLASS** | **FRAMES**    **NO FRAMES**     **All Classes** |  |
| SUMMARY: NESTED \| FIELD \| CONSTR \| METHOD | DETAIL: FIELD \| CONSTR \| METHOD |  |


---
